# The MuSeS project: a mixed methods study to increase understanding of the role of settlement and multicultural services in supporting migrant and refugee women experiencing violence in Australia

**DOI:** 10.1186/s12914-018-0184-0

**Published:** 2019-01-07

**Authors:** Cathy Vaughan, Yara Jarallah, Adele Murdolo, Linda Murray, Regina Quiazon, Karen Block, Lana Zannettino

**Affiliations:** 1 Center for Health Equity, Melbourne School of Population and Global Health, The University of Melbourne, Parkville, Victoria 3010 Australia; 2 Multicultural Centre for Women’s Health, Suite 207, Level 2, 134 Cambridge St, Collingwood, Victoria 3066 Australia; 3 School of Medicine, Faculty of Health, University of Tasmania, Hobart, Tasmania 7005 Australia; 4 College of Nursing and Health Sciences, Flinders University, Adelaide, South Australia 5001 Australia

**Keywords:** Violence against women, Family violence, Migrant, Refugee, Qualitative methods, Quantitative methods, Intersectionality, Multicultural services, Settlement services, Australia

## Abstract

**Background:**

Violence against women is a major human rights and public health issue globally. The experience of violence affects women across Australia, including the large number of migrant and refugee women who permanently or temporarily resettle in the country. Many women who experience violence find it difficult to access support, and evidence suggests women who have resettled in Australia face additional barriers to violence-specific services. Previous research, however, indicates many migrant and refugee women experiencing violence have contact with, and may disclose violence to, settlement and multicultural services. There has been limited research documenting current knowledge of, and practices by, settlement and multicultural services in relation to violence. The MuSeS project will address this knowledge gap and identify strategies settlement and multicultural services can use to better support women experiencing violence.

**Methods:**

This mixed methods research project will be conducted in six geographic communities across three Australian states: South Australia, Tasmania and Victoria. The different migration and resettlement patterns seen in these jurisdictions will enable generation of data relevant to settings across the country. The project has been designed in consultation with partner organisations from the settlement and multicultural service sector to ensure the research addresses their concerns and priorities. A mix of quantitative and qualitative methods will be used to generate rich data to inform strategies for settlement and multicultural services to better support women experiencing violence. These methods include an anonymous online survey of settlement and multicultural service providers to assess current knowledge, practices and professional development needs; in-depth interviews with settlement, multicultural and specialist (refugee) mental health service providers; in-depth interviews with refugee women; and focus group discussions with frontline workers and volunteers working with settlement and multicultural services.

**Discussion:**

Findings from this two-year research project will generate an in-depth understanding of the current and potential role of Australian settlement and multicultural services in supporting migrant and refugee women experiencing violence, and inform strategies to strengthen services’ capacity to appropriately respond. Given the prevalence of violence against women globally, findings will be useful for services engaging with migrant and refugee populations around the world.

## Background

As with Australian-born women, migrant and refugee women now living in Australia are known to experience high levels of family, sexual and other forms of violence [1, 2]. While many women who experience violence find it difficult to access support, there is substantial evidence to suggest that women who have resettled in Australia face a range of additional barriers that undermine access to violence-specific services [3–9]. These include women’s visa status, language and other communication barriers, lack of awareness of legal rights and of violence-specific services, reluctance to engage with authorities, and social isolation. Migrant and refugee women report that violence-specific services are not always able to provide them with the kinds of support they need, particularly if they are in an ongoing relationship with the perpetrator [10, 11]. Migrant and refugee women engaging with violence-specific services, including law and justice services, may receive support and assistance in maintaining their safety, but may also experience community ostracism and other forms of backlash that greatly compromise their health and wellbeing and that of their children [4, 5]. Therefore, it is important that research informs the development of strategies and pathways to support for migrant and refugee women who may find non-violence-specific services more appropriate, or safer to access, than mainstream domestic or family violence services. To date, there has been limited research undertaken to document current knowledge of, and practices by, workers and volunteers from Australian settlement and multicultural services in relation to violence against women and family violence.

Migrant and refugee women who have resettled in Australia can access a range of settlement and multicultural service providers in their communities. These include organisations delivering services funded by the Australian Government through the Department of Social Services, such as the Humanitarian Settlement Program (including the Adult Migrant English Program) and the Settlement Grants program; by the Department of Education and Training (such as the Skills for Education and Employment program); and by the Department of Health (such as the Program of Assistance for Survivors of Torture and Trauma). Depending on their visa type, migrant women and women seeking asylum may not be able to access some or all of the government funded programs listed above. Migrant and refugee women, and women seeking asylum, often engage with other multicultural services providing various forms of support in their communities, such as ethno-specific women’s groups, groups aiming to build vocational skills and microenterprise, and groups of (often) volunteers who provide material and practical support.

In recent years there has been increased interest in reaching and supporting migrant and refugee women experiencing violence through organisations providing settlement and multicultural services. Individual organisations working with migrant and refugee communities have declared their commitment to a strengthened response to family violence and have developed resource materials and pilot initiatives. Government agencies have also supported training programs. However, there is limited research evidence as to how settlement and multicultural services and service providers can best support women experiencing violence or implement early intervention initiatives.

The MuSeS (Multicultural and Settlement Services supporting women experiencing violence) project will build on our previous research with migrant and refugee women from over twenty countries [3, 4]. This research has documented the nature, dynamics and context of family violence against migrant and refugee women, explored women’s help seeking, and the appropriateness and effectiveness of violence-specific service responses. We have previously found that action was required to strengthen links between violence-specific services and agencies primarily working with migrant and refugee populations, such as settlement and multicultural services [3, 12]. Our previous research has also highlighted the need to engage with specialist refugee mental health services (a specific sub-set of settlement and multicultural services) to increase understanding about the interaction between refugees’ mental health and experiences of family violence. Up to 40% of refugees have experienced torture and trauma [13], and evidence is needed about how these experiences interact with violence perpetrated against refugee-background women, and the potential to better support refugee-background women experiencing family violence through specialist settlement and multicultural agencies working in this area (in Australia, members of the Forum of Australian Services for Survivors of Torture and Trauma).

This project will address these research gaps by developing an in-depth understanding of the current and potential role of settlement and multicultural services in supporting migrant and refugee women experiencing violence. The project will include a nested sub-study that specifically focuses on the interaction between the mental health of refugee women and family violence, and the opportunities for specialist services for survivors of torture and trauma to support refugee women experiencing family violence. The project will primarily collect data in South Australia, Tasmania and Victoria, but the evidence generated is of national relevance.

### Theoretical framework

The project will work within an intersectional feminist framework with the understanding that migrant and refugee women experience multiple and interlocking forms of oppression [14] and that gender alone is not a sufficient lens through which to view migrant and refugee women’s experiences of family violence and access to support [15, 16].

Migrant and refugee women’s experiences of family violence are situated at the intersection of conditions linked to social constructions of gender, race, sexuality, ethnicity, disability, family and class. As a result, migrant and refugee women’s experiences of domestic and family violence, and of help-seeking, can manifest in different ways due to the confluence of individual circumstances, life experiences and policy, social and legal contexts [3, 4, 9, 15, 16]. These contexts include, but are not limited to, experiences of racism, discrimination, social isolation, precarious visa status and the absence of citizenship rights, and prior experiences of war and persecution.

Migrant and refugee women’s lives are shaped by the intersection of relations of power that arise from these multiple social and identity positions, and the structural and social disadvantages these positions carry. Recognition of these dynamics of power and social inequalities informs our analysis of how settlement and multicultural services can best respond to women experiencing violence, and reinforces that a ‘gender lens’ is necessary but not sufficient [3, 15, 16].

## Methods/design

The overall aim of the MuSeS project is to generate evidence about how women experiencing violence can be better supported by settlement and multicultural services. Specific research questions include:

1. How do settlement and multicultural services currently support women experiencing violence?
2. What factors strengthen or undermine the capacity of settlement and multicultural services to provide support, implement early intervention initiatives, and to understand and respond to the interactions between refugees’ mental health and refugee women’s experiences of violence?
3. What opportunities are there for the delivery of effective interventions to support women experiencing violence through settlement and multicultural services?

The research team includes co-investigators from the University of Melbourne, the University of Tasmania, Flinders University and the Multicultural Centre for Women’s Health, with project partners including the non-government organisations AMES Australia (all states), Foundation House (Victoria), the Australian Migrant Resource Centre (South Australia) and the Australian Red Cross (Tasmania). The project has been designed in consultation with these partner organsiations, and following consultation workshops in each state with stakeholders from a range of settlement, multicultural and violence response services. These workshops were designed to explore the current and past experiences of the sector in relation to responding to women’s experiences of violence, and to document the priorities and concerns of the sector in relation to the research. The project is also supported by three Advisory Groups (one in each state), with Advisory Group meetings directly contributing to the overall research design and the development of data collection tools.

The research project will be conducted in six geographic communities across three states: South Australia, Tasmania and Victoria (two sites per state). The sites include communities with large and/or rapidly growing migrant and refugee populations, where there is a relative (for the state) density of settlement and multicultural service providers. These sites will enable us to generate information relevant to working with established and with newly-arrived migrant and refugee communities, and to services working in regional centres, metropolitan settings and in the rapidly expanding fringes of major cities. The different migration and resettlement patterns of each site will enable us to generate nationally relevant data.

A mix of quantitative and qualitative methods will be used to generate rich data relevant to the development of strategies that settlement and multicultural services can use to better support women experiencing violence. These methods include:

- an anonymous online survey of settlement and multicultural service providers to assess current knowledge, practices and professional development needs;
- in-depth interviews with settlement, multicultural and specialist mental health service providers;
- in-depth interviews with refugee women; and
- focus group discussions with frontline workers and volunteers working with settlement and multicultural services.

### Online survey

At least 500 staff and volunteers working in settlement and multicultural services will be invited to participate in an anonymous, online survey, with a minimum sample of 300 anticipated. Organisations working in the settlement and multicultural service sector in each Australian state or territory will be identified through co-investigator, research partner and Advisory Group networks. An email will be sent to the directors and/or research and policy managers at these organisations asking them to disseminate to their workforce and volunteers an invitation to participate in the online anonymous survey.

The survey will collect data about providers’ own practices and professional development needs. It has been designed to provide quantitative and qualitative information about:

- service providers’ knowledge about violence against women and about what family violence (in particular) is;
- whether violence against migrant and refugee women is an issue that service providers encounter in their day-to-day work, if so how frequently, and their response when they do receive a disclosure of violence;
- whether their organisation records disclosures, referrals or other relevant information in monitoring and information systems;
- whether the respondent has had any training relevant to violence against women; and
- whether they perceive that violence-related professional development and support would be beneficial (and if so, what kinds of support would be most effective).

Questions have been based on the recent workforce census undertaken by Family Safety Victoria and the National Community Attitudes towards Violence Against Women Survey [17, 18]. It is anticipated that the survey will take approximately 15 minutes to complete. It requires potential participants to ‘opt in’ online and is anonymous.

Sites for collection of qualitative data.

The project will primarily recruit participants from the local government areas of the City of Brimbank and City of Hume in Victoria (both sites in metropolitan Melbourne, and including suburbs on the rapidly growing urban fringe); the Cities of Hobart, Glenorchy and Launceston in Tasmania (all classified as regional in the Australian context, though they include the state capital); and the City of Port Adelaide Enfield and City of Salisbury in South Australia (both sites including suburbs in northern Adelaide, some of which are considered ‘regional’). These sites are home to a range of migrant communities, including communities that have primarily arrived in Australia as skilled migrants and communities whose members have primarily arrived as refugees or to seek asylum.

### In-depth interviews with service providers

Between 37 and 42 settlement and multicultural services providers (up to 14 per state) will be invited to participate in in-depth interviews. Organisations working in the settlement and multicultural sector in each state will be identified through co-investigator, research partner and Advisory Group networks, with representatives from many organisations already having participated in consultative workshops. This will include professionals providing diverse settlement and multicultural services including settlement case management; English language training; refugee health services, including specialist refugee mental health services; immigration legal advice; and social services. Interviewees will include women and men and will be conducted in English.

Service providers participating in in-depth interviews will be asked questions exploring:

- their perceptions of the role that settlement and multicultural services may play in supporting women experiencing violence;
- barriers to provision of violence-related support, including identification of factors that undermine effective referral; and
- opportunities that may exist for provision of support through the settlement and multicultural sector.

Interviews will also examine the capacity of organisations to provide trauma-informed support to women experiencing violence and explore workers’ capacity development needs in depth. These interviews will enable researchers to investigate the ways services currently provide support to women experiencing violence, including women in an ongoing relationship with the perpetrator. Researchers will also ask about how participants’ organisations work to prevent violence against women.

Participants involved in provision of specialist mental health services to refugees will be asked additional questions exploring their perceptions of:

- interactions between refugees’ mental health and experiences of family violence;
- the impact of mental illness (including, but not limited to post-traumatic stress disorder, anxiety and depression) on relationships within refugee families; and
- the potential for prevention and early intervention strategies to reduce family violence.

In-depth interviews with service providers are anticipated to last approximately 60 min and will be held at mutually convenient times and in private, quiet locations.

### Focus group discussions with volunteers and ‘frontline’ workers

Settlement and multicultural services engage a large voluntary workforce. Volunteers may be from a migrant or refugee background themselves, and provide a range of support to migrant and refugee families. They often work with clients over extended periods of time and may visit clients in their homes (for example, to provide tutoring). ‘Frontline’ workers include those staff of settlement and multicultural services who work directly and daily with migrant and refugee clients, providing services such as orientation to local health, education and social security systems. Frontline workers may accompany newly arrived individuals and families to appointments, and introduce them to cultural and religious networks. The rapport developed between frontline workers or volunteers, and clients accessing settlement and multicultural services, is such that they may receive disclosures of violence from members of migrant and refugee communities or otherwise learn about the occurrence of violence.

Volunteers and frontline workers will be provided information about the project through settlement and multicultural services’ newsletters or contacted through organisational email lists or other mass communication channels. Those interested will be invited to participate in a focus group discussion (FGD).

Approximately 90 volunteers and frontline workers will be recruited to participate in one of up to 15 FGDs (up to five in each state, with approximately six participants per group). Participants will include women and men. FGDs with participants working primarily with migrants will be held separately to FGDs with participants working primarily with refugees, given the likely different barriers and opportunities for responding to violence that are associated with women’s immigration status. FGDs will preferentially be conducted in English, however, we recognise that where groups are made up of participants all speaking the same language other than English (which may be the case for groups of workers and volunteers in some geographic locations), participants may prefer to hold the discussion in a language other than English. In such an instance, and depending on the language, the FGD will be facilitated by a bilingual member of the research team or a trained bilingual health educator.

Focus group discussions with volunteers and frontline workers will explore:

- participants’ experiences working with settlement and multicultural services;
- the types of violence-related support they believe would be most helpful for their clients;
- when people are most likely to seek support;
- barriers to community members seeking help for violence-related issues from the sector; and
- the training and ongoing support needs of volunteers and frontline workers.

Focus group discussions are anticipated to take up to 90 min, are likely to be conducted in English (as most groups will include participants speaking a mix of other languages) and will be conducted at convenient times and locations for participants.

### In-depth interviews with women with refugee backgrounds

Up to 30 women with refugee or refugee-like backgrounds (such as women seeking asylum) will be invited to participate in in-depth interviews. Participants in these interviews will be recruited through the networks of services providing specialist mental health and other health services in each state. In-depth interviews with refugee women will explore:

- their perceptions and experiences of receiving support for family violence from a settlement or multicultural service;
- their perceptions about the interaction between mental health and family violence; and
- the impact of mental illness of family relationships, help seeking and access to services.

Interviews are anticipated to take approximately 60 min and will be held in quiet and private spaces at organisations already familiar to the participant (e.g. partner organisations’ interview rooms).

Women currently receiving therapeutic care for the sequelae of torture or trauma or for mental illness will be excluded from participation in the study. Similarly, women who are assessed by service providers and/or the research team as being in the middle of a current crisis or any situation that could be aggravated by involvement with the research will also be excluded for safety reasons. Interviews will not be conducted with more than one member of the same household.

### Analysis and sample size

Quantitative data from the survey will be analysed using descriptive statistics, with proportions with confidence intervals calculated for categorical variables and means, medians, interquartile ranges and ranges for continuous variables. A non-probability sample size of 300 survey respondents will allow us to detect a proportion of 50%, with 95% confidence intervals of 44, 56%. Qualitative survey data describing service providers’ knowledge about family violence, current practices and level of training and support will be analysed thematically as described below for interview transcripts.

Prior experience suggests the indicative sample sizes for the qualitative components as listed will enable us to achieve thematic saturation within the data. All interviews and group discussions will be recorded with participant permission; in addition, notes will be taken by the interviewer or by the FGD note-taker. Permission to audio-record will be included in the consent form, as a requirement for participation. Audio-recordings will be transcribed and translated where necessary. Where sound quality precludes accurate transcription (as is sometimes the case with FGD recordings), researchers’ notes will be written up in detail to include in the analysis, with the origin of data noted during analysis.

The research team will draw on an intersectional feminist conceptual framework in analysing the qualitative data [15]. Initially the team will develop a draft list of themes and codes, based on our research questions and concepts drawn from the literature on violence against migrant and refugee women. Following detailed review of transcripts, the team will also work to identify codes from the empirical material and cluster these into themes using a well established approach to thematic analysis [19]. Codes will be organised into overarching themes, and a draft coding framework developed. All data will be coded into the identified themes using NVivo software and analysed for variations and patterns present in relation to the research questions. Qualitative data will be coded by members of the research team in each state, with the first author (Principal Investigator) also coding a selection of data to assess the draft framework for inter-coder reliability.

After coding and initial analysis, consultative data analysis workshops will be conducted in each state to give Advisory Group members and other stakeholders the opportunity to interrogate and provide feedback on our early analysis. This feedback will be incorporated into subsequent analysis and the project’s final report.

### Participant and researcher safety

Refugee women who have experienced violence have already been exposed to numerous risks to their wellbeing, and can experience further harms as a result of their participation in research [20]. Workers providing services to migrants and refugees, in often under-resourced and politically contentious circumstances, can experience high levels of work related distress. Researchers engaging with women about their experiences of violence, or engaging with particularly stressed service providers, can also experience vicarious trauma [21]. A comprehensive safety protocol has been developed to support the research team in managing risks for participants and researchers that may be associated with the MuSeS project, with a safety monitoring process to be implemented throughout to ensure the safety of participants and research team memembers.

The project will take a trauma-informed approach [22], with all research team members trained to recognise and respond appropriately to women sharing their experiences of violence. Team members also recognise that the mental health impacts of refugees’ previous exposure to traumatic events prior to resettlement may increase the likelihood that participants with a refugee background may experience intense emotions through the research process [23]. In-depth interview and FGD questions have been carefully designed to minimise the risk of participants becoming distressed.

### Other ethical considerations

All participants will be informed about the study using a plain language statement which will be available in English. Any participants who are unable to read a plain language statement in English, will have the language statement read to them in a language they understand by a bilingual member of the research team, a bilingual health educator or interpreter. All participants will be 18 years of age or older, with the ability to provide independent voluntary consent. Adult participants who are unable to give informed consent will be excluded. The MuSeS project has been granted ethics approval by the University of Melbourne Human Research Ethics Committee (ethics ID 1852384), the Tasmanian Social Sciences Human Research Ethics Committee (ethics ID H0017650) and the Flinders University Social and Behavioural Research Ethics Committee (ethics ID 1852384).

## Discussion

In Australia, 37% of women have experienced physical or sexual violence since the age of 15, with one in six women having experienced violence by a current or previous partner since the age of 15 [24]. Australia is a settler colonial state with one of the most multicultural societies on earth; over 28% of the population were born overseas [25]. All current evidence suggests that the large number of migrant and refugee women living in Australia experience violence at rates at least as high as the wider population [1, 24], and that they face particular and additional barriers in accessing specialist violence services. Migrant and refugee women may, instead, disclose violence to and seek help from settlement and multicultural services [3]. There has been limited research documenting current knowledge and practices of workers and volunteers from settlement and multicultural services, or their capacity development needs, in relation to family violence and violence against women. The current evidence base is insufficient to inform settlement and multicultural services about how they can most effectively support women experiencing violence. MuSeS will make a unique contribution to the literature by generating evidence based on the perspectives of volunteers and workers with diverse experiences in the settlement and multicultural service sector. This evidence can underpin initiatives in the settlement and multicultural service sector to better support women experiencing violence.

Forty percent of refugees have experienced torture and trauma [13], and yet few research projects exploring violence against migrant and refugee women in Australia have examined the interaction between the mental health of refugee women and vulnerability to family violence. MuSeS will increase our understanding of how traumatic experiences interact with violence perpetrated against women with refugee backgrounds. Knowledge generated will have a particular focus on how settlement and multicultural services, including specialist mental health services (such as those provided through members of the Forum of Australian Services for Survivors of Torture and Trauma), could support women experiencing family violence.

Project findings will also contribute to the fledgling evidence base about the intersectional effects of policies relating to migration, settlement and violence against women (including its prevention, early intervention and response). An increased understanding of the interaction of women’s experiences and services’ current capacities and ways of working will provide a benchmark for the development of ‘good’ intersectional policy and evidence-informed advocacy in relation to supporting migrant and refugee women.

The findings will be widely communicated in project reports and publications and will include practical recommendations for practice, and legislative and policy change, that can be taken up by a range of stakeholders including ethno-specific and multicultural organisations and community advocates.

The engagement and participation of volunteers is a critical aspect of the project, and their involvement will promote recognition of the invisible work that volunteers do in the community. In addition to drawing upon volunteers’ expertise and insights about migrant and refugee women’s needs, data collected during the focus group discussions will also produce valuable evidence about the professional development and support needs of volunteers themselves. This is particularly critical evidence, as volunteers are a group upon whom the settlement and multicultural service sector entirely depends and yet one that is often neglected in policy and practice reform. Given that many volunteers working in multicultural and settlement services are also migrants or refugees, project findings will contribute to the scant evidence-base for the valuable contribution a bicultural, bilingual workforce can make to social and community outcomes.

The mix of quantitative and qualitative data generated by MuSeS will provide policy makers and service providers in the settlement and multicultural service sector with a robust evidence base for the design and delivery of effective interventions to build worker capacity in relation to violence against women, and to provide effective support to migrant and refugee women experiencing violence.

## Abbreviations

FGD: Focus Group Discussion
MuSeS: Multicultural and Settlement services Supporting Women experiencing violence
